# Prognostic value of the BRAF V600E mutation in papillary thyroid carcinoma

**DOI:** 10.3892/ol.2013.1713

**Published:** 2013-11-29

**Authors:** GUOPING HE, BAOJIAN ZHAO, XU ZHANG, RIXIANG GONG

**Affiliations:** 1 The Center for Reproductive Medicine, Anhui Provincial Hospital Affiliated to Anhui Medical University, Hefei, Anhui 230001, P.R. China; 2 Molecular Pathology Center of the General Hospital of the Air Force PLA, Beijing 100081, P.R. China; 3 Department of Thyroid and Breast Surgery, West China Hospital, Sichuan University, Chengdu, Sichuan 610041, P.R. China

**Keywords:** BRAF V600E, prognostic value, papillary thyroid carcinoma

## Abstract

The aim of the present study was to investigate the prevalence of the BRAF V600E mutation in papillary thyroid carcinoma (PTC) and to determine the correlation between this mutation and indicators of poor prognosis and outcome in patients with PTC. The BRAF V600E mutation status was analyzed in 187 tumor samples using the multiplex allele-specific PCR method. Univariate and multivariate analyses were performed to investigate the association of the BRAF V600E mutation with clinical features and patient outcome. The sensitivity of the multiplex allele-specific PCR combined with denaturing high-performance liquid chromatography reached ~1%. The BRAF V600E mutation was observed in 63.6% (119/187) of tumor tissues, predominantly in PTC specimens, and no BRAF mutation was identified in other benign-type thyroid diseases. The univariate analysis indicated that the BRAF V600E mutation was associated with age, tumor stage and prognosis (P<0.05). In addition, the frequency of the BRAF V600E mutation was significantly different in the central (75.3%) and lateral neck (49.3%) lymph nodes of patients with lymph node metastasis. Multivariate logistic regression analysis showed that the BRAF V600E mutation (HR, 2.471; 95% CI, 1.149–5.312) and lymph node metastasis (HR, 3.003; 95% CI, 1.027–8.771) are independent factors that predict tumor prognosis. Thus, the BRAF V600E mutation is an independent risk factor that may be used to predict thyroid cancer persistence/recurrence.

## Introduction

Papillary thyroid carcinoma (PTC) is a common endocrine tumor with an incidence that has increased over recent decades in China. PCT exhibits a high cure rate with 10-year patient survival rates estimated at 80–90% (1). Currently, surgery remains the predominant treatment option for PTC; however, even following curative resection, 20–30% of PTC patients experience recurrence and/or metastasis, which is associated with increased mortality (2). Numerous clinical and pathological factors, including age, gender, tumor size, histological type, tumor interstitial fibrosis and extrathyroidal infiltration, have been shown to have limited prognostic value in PTC (2–7).

In recent years, the study of the molecular mechanisms of PTC has demonstrated that the BRAF gene mutation is a significant event in the process of this disease. The V600E mutation of the BRAF gene was identified in 28–83% of PTC specimens, however, no mutations were observed in normal thyroid tissue and tissue from patients with benign thyroid disease (4,8–11). A large number of studies have demonstrated that the BRAF gene mutation is associated with pathological features, including patient age, extrathyroidal invasion, lymph node metastasis and tumor stage, which aid in determining patient prognosis (5,6,12–14). In the present study, multiplex allele-specific PCR technology combined with denaturing high-performance liquid chromatography (DHPLC) was established to investigate the correlation between the BRAF gene mutation and the clinical features, pathological diagnosis, treatment and prognosis in 187 patients with PTC. The BRAF V600E mutation was observed in PTC, while no BRAF mutations were identified in benign-type thyroid diseases. In addition, significant correlations were observed between the BRAF mutation and age (>45 years), tumor-lymph node-metastasis stage and tumor persistence/recurrence.

## Patients and methods

### Patients

To be eligible for the study, patients were required to present with pathologically confirmed PTC according to the tumor-lymph node-metastasis classification system of the International Union against Cancer and the American Joint Committee on Cancer. Tumor samples and 20 adjacent normal tissues were obtained from 187 patients in the West China Hospital, Sichuan University (Sichuan, China) between May 2009 and May 2011 for use in this study. The study was reviewed and approved by the West China Hospital, Sichuan University. Written informed consent was obtained from the patients. Thyroid cancer K1 cells with the BRAF V600E mutation and RO82-W-1 cells with wild-type BRAF were obtained from the Molecular Biology Laboratory of the West China Hospital. All patients underwent total or near-total thyroidectomy. Patients were then routinely observed as outpatients, typically every 6–12 months, and were evaluated for cancer persistence/recurrence with serum thyroglobulin (Tg) testing during the 2-year follow-up. For all cases, persistent/recurrent PTC was defined as a detectable basal serum Tg level (>1 ng/ml) (9). A total of 176 patients completed follow-up.

### DNA extraction

Genomic DNA was isolated from formalin-fixed parafin-embedded tissue sections with a Qiagen TIANamp genomic DNA kit (Qiagen, Hilden, Germany) and from cells lines with a Tiangen genomic DNA extraction kit (Tiangen Biotech, Co., Ltd., Beijing, China), according to the manufacturer’s instructions. DNA was analyzed by agarose gel electrophoresis and a UV photometer (UVP, Upland, CA, USA).

### PCR primers

The BRAF mutation primers (BRAF mut) were designed based on the BRAF sequence, with a mismatched nucleotide at the 3′ end, so that wild-type BRAF was not amplified by these primers. The length of the PCR products was 126 bp. The BRAF sequencing primers (BRAF seq) were also designed. In addition, TBXAS1 was chosen as a reference gene. Information concerning these primers is listed in Table I.

### Multiplex PCR amplification

BRAF and TBXAS1 genes were amplified in the same 25-μl amplification system, which included 30 ng DNA templates, 10 pmol BRAF primers, 5 pmol TBXAS1 primers, 0.1 mol/l dNTPs and 1.5 units *Taq* enzyme. The thermal cycling protocol for PCR involved an initial denaturation step at 95°C for 5 min followed by 30 cycles at 94°C for 30 sec, 54°C for 30 sec and 72°C for 30 sec. The BRAF gene was amplified in a 50 μl amplification system for direct sequencing.

### DHPLC

DHPLC was performed using the Transgenomic Wave Nucleic Acid Fragment Analysis system with a DNASep column (Transgenomic, Omaha, NE, USA). The mobile phases comprised 0.05% acetonitrile in 0.1 M triethylammonium acetate (TEAA; eluent A) and 25% acetonitrile in 0.1 M TEAA (eluent B). The PCR products were denatured at 95°C for 5 min and then cooled to 50°C to allow the formation of heterozygote DNA. A 0.9-ml/min flow rate was used, and the ultraviolet detector was set at 260 nm. The results were analyzed by Navigator software (Transgenomic, Omaha, NE, USA). The heterozygous profiles were investigated by visual inspection of the chromatograms on the basis of the appearance of additional later-eluting peaks. Corresponding homozygous profiles showed only one peak. To determine the detection limit of DHPLC, DNA was extracted from the RO82-W-1 (BRAF wild-type) and K1 (BRAF V600E mutation) cells. Serial mixtures (BRAF V600E/Total DNA, 50, 25, 10, 3 and 1%) were made for the DHPLC analysis.

### Statistical analysis

Statistical analysis was performed using SPSS 13.0 statistical software (SPSS, Inc., Chicago, IL, USA) and a χ^2^ test was used for the comparisons. The category data were estimated by odds ratio (OR) and 95% confidence intervals (CI) in a meta-analysis. P<0.05 was considered to indicate a statistically significant difference.

## Results

### Clinical data

A total of 187 cases of PTC, consisting of 159 females and 28 males (age, 8–80 years; mean ± SD, 42.57±12.88), were selected for systematic analysis. Among them, 59 patients presented with bilateral leaf papillary carcinoma, 47 with PTC of the left lobe, 78 with PTC of the right lobe and three with papillary carcinoma of the thyroid isthmus.

### Multiplex allele-specific PCR sensitivity detection

**Figure 1:**
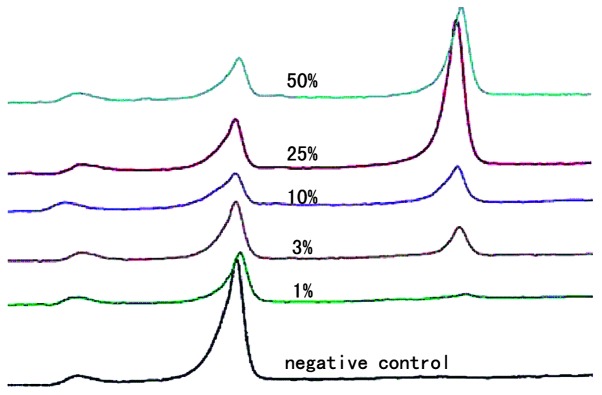
Results of multiplex allele-specific PCR detection of BRAF V600E mutation sensitivity. 1–50%, different concentrations of the positive control; negative control, RO82-W-1 cells.

In order to investigate the sensitivity of multiplex allele-specific PCR combined with DHPLC, different concentrations of V600E mutation PCR products (1, 3, 10, 25 and 50%) were detected by this method. According to the DHPLC results, two elution peaks were observed in the presence of a V600E mutation (Fig. 1); the right elution peak was due to the BRAF mutation and the left was due to the internal reference TBXAS1 gene. It was demonstrated that multiplex allele-specific PCR detection sensitivity may be up to 1% (Fig. 1).

### Prevalence of BRAF V600E mutation in PTC samples

**Figure 2:**
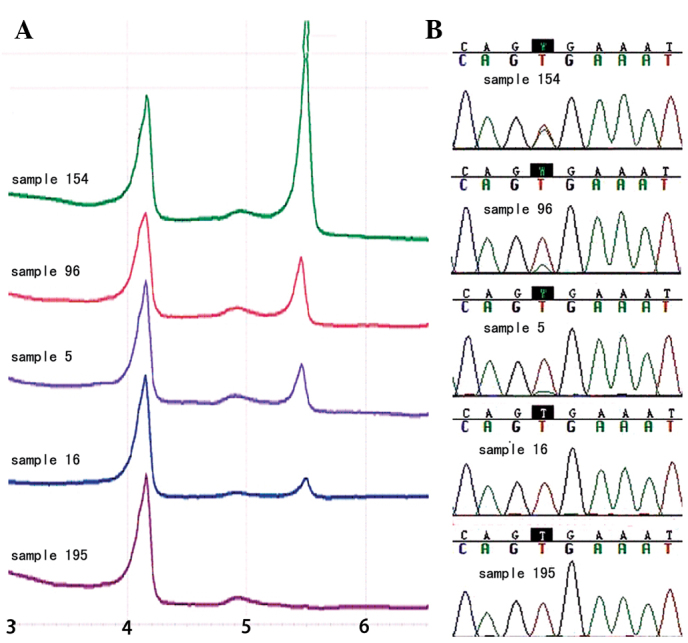
Representative results of denaturing high-performance liquid chromatography (DHPLC) and sequencing for BRAF V600E mutations. (A) BRAF V600E mutations were detected in several samples using multiplex allele-specific PCR. A low level mutation peak was observed in sample 16. (B) BRAF V600E mutations were detected by direct sequencing; a low level mutation was observed in sample 5 and no mutation was identified in sample 16.

The BRAF V600E mutation was detected in 63.6% (119/187) of PTCs (Fig. 2A). No mutation was identified in the 20 benign thyroid lesions (data not shown). In addition, these results were confirmed by direct sequencing (Fig. 2B).

### Association of BRAF V600E mutation status with PTC pathological features

In patients with conventional PTC, the BRAF V600E mutation was associated with age, tumor stage and prognosis (P<0.05). However, the frequency of the BRAF V600E mutation was not correlated with gender, tumor size, lymph node metastasis or location of the lesion (Table II). In addition, the BRAF V600E mutation was significantly different in the central lymph nodes and lateral neck lymph nodes (75.3 vs. 49.3%; P=0.002) of patients with lymph node metastasis.

### BRAF V600E mutation status as a prognostic factor in PTC

A correlation analysis of PTC diagnosis and clinical features was conducted. Based on the univariate analysis, tumor recurrence/metastasis was associated with tumor size and lymph metastasis, but not with gender, age and tumor stage. Furthermore, the multivariate logistic regression analysis showed that lymph node metastasis and BRAF V600E mutation were independent factors that predicted tumor prognosis (Table III).

## Discussion

BRAF V600E mutation detection in PTC demonstrated that the BRAF V600E mutation was present in ~63.6% of the tumor tissue samples, predominantly in those of PTC. In addition, no BRAF mutations were observed in other benign-type thyroid diseases, indicating that this genetic event was likely to be a key determinant of the papillary cancer phenotype.

The occurrence and development of PTC involves multiple genetic abnormalities, in which the BRAF V600E gene mutation is the most common variant and contributes to the destabilization of the kinase encoded by the gene. The majority of studies have demonstrated that the BRAF V600E mutation is common in PTC and that the frequency varies from 18 to 87%. Although certain studies have indicated that geographical and histological subtype classification factors may account for these differences, the reliability of the detection method must also be taken into consideration (7,15). In the present study, multiplex allele-specific PCR combined with DHPLC was used to detect the mutation. It was demonstrated that detection sensitivity may be up to 1% by this method, showing that this would be a reliable method of detection in clinical samples.

Although the prognostic value of the BRAF V600E mutation in PTC is controversial, several studies have observed an association between the mutation and a poor prognosis (5,13,16). To analyze this association in Chinese patients, 187 PTC samples were analyzed for the mutation and its association with clinical features. It was observed that the BRAF V600E mutation was associated with age, tumor stage and prognosis. In addition, the frequency of the BRAF V600E mutation was significantly different in the central and lateral neck lymph nodes of patients with lymph node metastasis, which is consistent with previous studies (14,17,18). However, the BRAF V600E mutation was not correlated with gender, tumor size, lymph node metastasis and location of the lesion.

Furthermore, the correlation of a PTC diagnosis and the clinical features were analyzed by univariate and multivariate logistic regression analyses. The results indicated that tumor persistence/metastasis was significantly associated with tumor size, lymph node metastasis and BRAF mutation in the univariate analysis. Concurrent with other studies, lymph node metastasis and BRAF mutation were independent factors in the prediction of tumor prognosis in the multivariate logistic regression analysis (Table III) (5,12,16,19,20).

In conclusion, the BRAF V600E mutations in PTC and the lymph nodes are independent factors in the prediction of tumor prognosis. Moreover, the BRAF V600E mutation is significantly different in central lymph nodes and lateral neck lymph nodes of patients with lymph node metastasis. Testing for this mutation may be useful for selecting initial therapy mode and for follow-up monitoring in PTC patients.

## Figures and Tables

**Table I:** Allele-specific PCR primer sequences for the BRAF gene.

Genes	Sequence	PCR products, bp
BRAF mut	Forward, 5′-GGTGATTTTGGTCTAGCTACATA-3′	126
	Reverse, 5′-GGCCAAAAATTTAATCAGTGG-3′	
TBXAS1	Forward, 5′-GCCCGACATTCTGCAAGTCC-3′	100
	Reverse, 5′-GGTGTTGCCGGGAAGGGTT-3′	
BRAF seq	Forward, 5′-CTCTTCATAATGCTTGCTCTG-3′	269
	Reverse, 5′-GAGACCTTCAATGACTTTCTAGTAAC-3′	

BRAF mut, BRAF mutation primers; BRAF seq, BRAF sequencing primers.

**Table II:** Correlation analysis between BRAF V600E mutation and clinical features.

		BRAF V600E mutation, %		
			
Clinical features	Cases, n	Positive	Negative	P-value
Gender				0.401
Male	28	20	8	
Female	159	99	60	
Age, years				0.002
>45	75	58	17	
<45	112	61	51	
Tumor size, cm				0.279
>2	144	95	49	
≤2	43	24	19	
Lymph node metastasis				0.582
N1	146	91	55	
N0	41	28	13	
Metastasis region				0.002
Central node	73	55	18	
Lateral node	73	36	37	
Tumor stage				
I and II	119	66	53	0.003
III and IV	68	53	15	
Unilateral or bilateral lesions				0.138
Bilateral	59	33	26	
Unilateral	125	85	40	
Lesion location				0.698
Left lobe	47	31	16	
Right lobe	78	54	24	
Prognosis				0.03
Normal	117	69	48	
Relapse/metastasis	59	45	14	

**Table III:** Associations between the prognosis of PTC and clinical features.

Clinical features	Univariate analysis P-value	Multivariate regression analysis		

		P-value	HR	95% CI
Gender	0.119	0.134		
Age, years	0.871	0.999		
Tumor size, cm	0.004	0.074	2.545	0.915–7.092
Lymph metastasis	0.003	0.044	3.003	1.027–8.771
Tumor stage	0.406	0.999		
BRAF mutation	0.030	0.021	2.471	1.149–5.312

PTC, papillary thyroid carcinoma; OR, odds ratio; CI, confidence interval.
